# Tremor measurements in a 22-year cohort study of workers exposed to hand-held vibrating tools

**DOI:** 10.1007/s00420-020-01612-8

**Published:** 2021-02-19

**Authors:** Rita Bast-Pettersen, Karl-Christian Nordby, Gunilla Wastensson, Lisa Aarhus

**Affiliations:** 1 Department of Occupational Medicine and Epidemiology, National Institute Of Occupational Health, Oslo, Norway; 2 Department of Occupational and Environmental Medicine, University of Gothenburg, Gothenburg, Sweden

**Keywords:** CATSYS Tremor pen^®^, Hand-held vibrating tools, Grooved Pegboard test, Free thyroxine (s-fT_4_), Glycated hemoglobin (HbA1c)

## Abstract

**Objectives:**

The objectives of this cohort study were to evaluate possible long-term effects of occupational exposure to hand-arm vibration (HAV) in terms of increased tremor. The aims were to evaluate whether exposure during follow-up, baseline hand-arm vibration syndrome (HAVS), baseline manual dexterity or current medical conditions or life-style habits might be associated with increased tremor. A further aim was to compare two different activation conditions: postural vs rest tremor.

**Methods:**

Forty men (current age: 60.4 years) who had previously worked as manual workers in a specialized engineering and construction company enrolled in the study. Their hand functions had been examined in 1994. At the baseline examination, 27 had been diagnosed with HAVS, while 13 were not exposed. The follow-up examination in 2016–2017 comprised the CATSYS Tremor Pen^®^ for measuring postural and rest tremor and the Grooved Pegboard Test for assessing manual dexterity. Blood samples were taken for assessing biomarkers that might have impact on tremor.

**Results:**

Neither cumulative exposure to HAV during follow-up nor HAVS at baseline were associated with increased tremor. A test for manual dexterity at baseline was significantly associated with increased tremor (Tremor Intensity) at follow-up. Blood markers of current medical conditions and tobacco consumption were associated with increased tremor. Rest tremor frequency was higher than postural tremor frequency (*p* < 0.001).

**Conclusions:**

The main findings of this 22-year cohort study were no indications of long-term effects on tremor related to HAV exposure and previous HAVS status. However, baseline manual dexterity was significantly associated with increased tremor at follow-up. Activation conditions (e.g., hand position) are important when testing tremor.

## Introduction

The use of hand-held vibrating tools can lead to hand injuries in terms of hand-arm vibration syndrome (HAVS), which is composed of vascular, neurological and muscular components (Burström et al. 2006; Heaver et al. 2011; Ye et al. 2015). HAVS is often diagnosed by clinical examination based on the Stockholm workshop scale (Gemne et al. 1987; Lawson 2016; Aarhus et al. 2018; Poole et al. 2019). A recent study by Vihlborg et al. (2017) reported that 21% of a group of examined workers with hand-arm vibration (HAV) exposure had vibration injuries. Subjects with HAVS can have neurological symptoms such as reduced sensory function, tingling and paraesthesia. The sensorineural component of HAVS is usually evaluated by scoring systems such as the Stockholm sensorineural system (Brammer et al. 1987), by vibrometry and by tests for manual dexterity, for instance, a pegboard test. However, it has been proposed that other forms of evaluation systems can sometimes be more appropriate (Griffin 2008).

### Tremor

Tremor is an involuntary, rhythmical, oscillatory movement of a body part (Findley 1996; Bhatia et al. 2018). The most important features of tremor are amplitude and frequency together with the activation condition (Deuschl et al. 1998). Physiological hand tremor is suggested to contain two distinct rhythmic components, a passive mechanical oscillation that depends on the part of the body from which it is recorded (mechanical reflex component) and a central neurogenic component (Elble and Koller 1990; Bhatia et al. 2018).

According to the classification proposed by the International Tremor Foundation (Findley 1996; Deuschl et al. 1998), updated in 2018 (Bhatia et al. 2018), rest tremor occurs when muscles are not voluntarily activated, and the body part is completely supported against gravity, while action tremor occurs with voluntary contraction of muscles. The latter includes postural, kinetic, task-specific, and isometric tremors (Bhatia et al. 2018). Thus, postural tremor, present while voluntarily maintaining a position against gravity, is classified as a kind of action tremor.

In a recent study of tremor among HAV-exposed workers, we found differences in several tremor parameters associated with the activation condition (rest tremor vs postural tremor) (Bast-Pettersen et al. 2017).

It has been suggested that disturbance in hand function due to HAV exposure could lead to increased tremor (Bast-Pettersen et al. 2017). However, few studies have examined tremor among vibration-exposed workers. Futatsuka et al. (2005) and Bylund et al. (2002) recorded tremor as subjective complaints but without quantitative measures in their studies of workers exposed to vibration. There are several tremor rating scales available, but many of them provide limited assessment of tremor, and commercially available transducers are regarded as suitable for quantitative assessment of tremor. In the 1998 consensus statement (Deuschl et al. 1998), recommendations for a tremor study design also advocated including measures of performance, for instance, a peg board test (Deuschl et al. 1998).

Edlund et al. (2015) applied an accelerometer, the CATSYS Tremor Pen^®^ (version 7.0, Danish Product Development 2000), in a study of 139 male workers exposed to HAV. They found no changes in quantitatively measured postural tremor parameters associated with either cumulative or current HAV exposure. Tobacco consumption and higher age were statistically significant predictors of increased tremor amplitude, although the age effect was only observed with the left hand.

In a cross-sectional study of 103 road maintenance workers examined with the CATSYS Tremor Pen, cumulative exposure to vibrating hand tools was associated with increased postural and rest tremor among smokers and users of smokeless tobacco. Postural tremor was more strongly associated with exposure than rest tremor. The subjects diagnosed with HAVS had increased postural tremor with a higher frequency than the subjects without HAVS (Bast-Pettersen et al. 2017).

Over a 22-year span, ongoing exposure may occur and new health conditions may emerge. Tobacco use and alcohol consumption may affect dopaminergic structures of the brain, and studies have shown increased tremor among smokers with various occupational exposures (Bast-Pettersen et al. 2004; Bast-Pettersen and Ellingsen 2005; Ellingsen et al. 2006; Bast-Pettersen et al. 2017). Several medical conditions have been associated with increased tremor, such as hyperthyroidism, hypoglycaemia and nonketotic hyperglycaemia in diabetic subjects (Aminoff and Josephson 2014).

### Objectives

The objectives of this long-term cohort study were to evaluate the following:

Whether HAV exposure during the 22-year follow-up period predicted increased tremor.

Whether HAVS at baseline predicted increased tremor 22 years later.

Whether a test for manual dexterity at baseline predicted increased tremor 22 years later.

Whether current medical conditions and life-style habits (tobacco and alcohol consumption) were associated with increased tremor.

Whether the activation condition (hand position) was related to tremor parameters.

## Subjects and methods

**Fig. 1:**
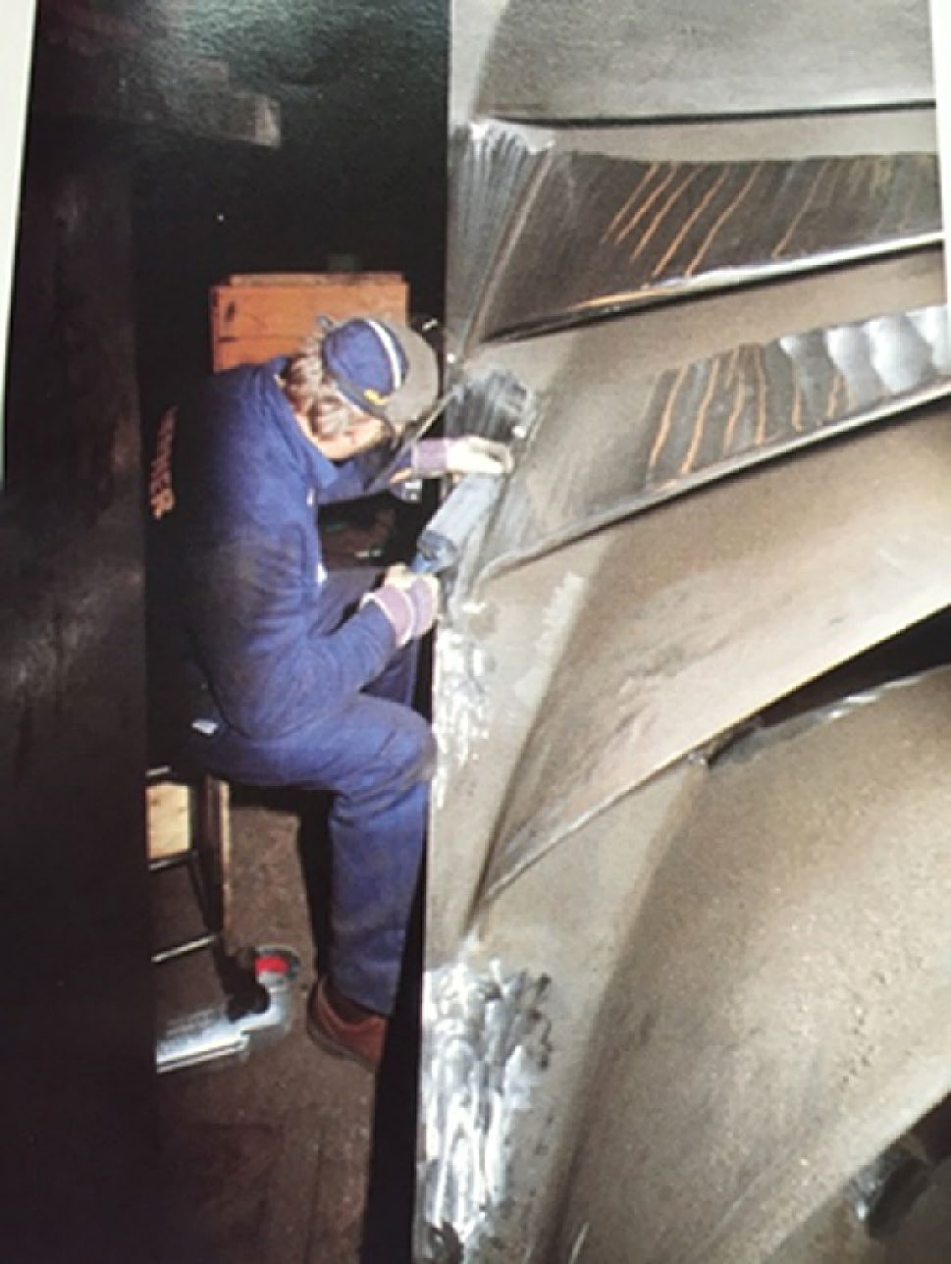
In this image from 1994, a worker is polishing the surface of a turbine wheel

In 1994, a physician employed in a specialized engineering and construction company (Fig. 1) was concerned about the many possible work-related injuries associated with working with vibrating hand tools. All employees (*n* = 211) in two departments of the company participated in a HAVS examination. Based on the responses to a questionnaire distributed in 1994, we selected workers for the 2016/2017 follow-up study. The following criteria were used: (1) The workers who were exposed to hand-held vibrating tools at work and who had evident symptoms [numbness in fingers and/or vibration-induced white finger (VWF) attacks] were classified as the HAVS group. The workers were explained the definitions of white finger attacs (“sharp” line of demarcation between normal and abnormal skin colour) and hand numbness. Thereafter they were interviewed using a questionnaire containing the questions: “During the last period of time, have you felt hand numbness?” and “During the last period of time, have you felt white finger attacks?”. (2) The workers who were not exposed to hand-held vibrating tools and who had no evident neurological or vascular symptoms were classified as the unexposed group (Aarhus et al. 2018, 2019).

One-hundred and ten workers met these criteria (68 in the HAVS group in 1994 and 42 in the unexposed group in 1994). As the company had been shut down in 1999, we did not have access to updated personal information. We used telephone directories to try to find several of the workers. This was the main reason for why 49 subjects could not be traced, among which two were documented not alive. The remaining 61 workers were sent an invitation letter. Among these 61 invited subjects, 40 participated in the 2016–2017 study (participation rate among the invited subjects: 66%). Twenty-one subjects did not respond, or they declined to participate: 11 of 38 in the group with HAVS in 1994 (participation rate: 71%) and 10 of 23 in the group without HAVS in 1994 (participation rate: 57%). These subjects declined for various reasons, including a long travel time or lack of time. At the time of the diagnosis in 1994, the 27 subjects in the HAVS group had been HAV-exposed for on average of 18.3 (SD 8.9) years.

All subjects volunteered to participate in the study, and their written informed consent was obtained. The study was approved by the Norwegian Regional Committee for Medical and Health Research Ethics, REK South-East, Norway.

### Examinations

The examinations took place in Oslo, Norway, in 1994 and 2016/2017. The 2016–2017 study was conducted during the cool season from September 2016 to March 2017. In 1994, the workers were tested with a test battery comprising the Grooved Pegboard test (Lafayette Instrument), a cold challenge photoplethysmography test (PPG) and vibrometry for testing sensory-neural thresholds (Aarhus et al. 2018, 2019). The subjects also underwent a clinical examination and an interview that included assessment with the Stockholm Workshop Scale. The same procedure was repeated in the follow-up study, but a tremor test and blood samples were added.

### Interview

We interviewed the workers about their work and leisure exposure to hand-held vibrating tools (type of tool, hours per day, days per year and number of years); smoking habits and the use of smokeless tobacco; medical conditions; medications; and neurological, vascular or muscular symptoms of HAVS. In addition to the self-reported consumption of alcohol beverages calculated as L pure alcohol/year (Hauge and Irgens-Jensen 1987), their current alcohol consumption was based on their levels of carbohydrate-deficient transferrin (CDT).

### Tremor test

The CATSYS Tremor Pen^®^ (version 7.0, Danish Product Development 2000) was used to measure postural and rest hand tremor. The test equipment consists of a biaxial micro-accelerometer that is embedded in a low-mass stylus (12 cm × 0.8 cm) and connected to a data logger. Tremor is recorded in a frequency band ranging from 0.9 Hz to 15 Hz. The combined signal from the two perpendicular accelerometers is transformed by the system’s software using fast Fourier transformation. The CATSYS software provides four measures: Tremor Intensity, Center Frequency, Frequency Dispersion and Harmonic Index. The magnitude or strength of the tremor is denoted by the Tremor Intensity (amplitude) measure, and in the present study, we used this measure to analyse the association between the predictors and the magnitude of tremor.

The Tremor Intensity, the Center Frequency and the Frequency Dispersion were included in an analysis, where postural tremor was compared with rest tremor. The Harmonic Index was omitted in the comparison between postural tremor and rest tremor, since it has been suggested to be unreliable in test–retest experiments (Edwards and Beuter 1999; Bast-Pettersen and Ellingsen 2005; Wastensson et al. 2016).

The method has previously been extensively described (Despres et al. 2000; Bast-Pettersen and Ellingsen 2005; Wastensson et al. 2012; Wastensson et al. 2016; Bast-Pettersen et al. 2017). The testing time was set to 16.4 s, which is longer than the default test time of 8.2 s but has now been applied in several studies (Wastensson et al. 2012; Ellingsen et al. 2014; Wastensson et al. 2016; Bast-Pettersen et al. 2017).

Postural tremor was examined, while the subject was sitting in a chair without an armrest and was required to hold the Tremor Pen as an ordinary pen in front of the navel with the elbow bent at an angle of 90° and free of any contact with the body or other support. Rest tremor was examined with the Tremor Pen taped to the hand in the same position as one would hold an ordinary pen, while the arm was resting on the table, and the subject was asked to relax as much as possible during the test session (Bast-Pettersen et al. 2017).

### Manual dexterity

*The Grooved Pegboard Test* (Lafayette Instrument Company^®^), a test of manual dexterity and motor speed, was applied to assess the sensori-neural part of HAVS. The test consists of a small board with a 5 × 5 set of slotted holes angled in different directions and 25 pegs with a ridge on one side. The score was the time to completion in s for each hand.

The same certified clinical neuropsychologist (RB-P) conducted the testing of all subjects.

### Collecting biological samples and determining biomarkers

No blood samples were taken in the baseline study. In the follow-up study, biological samples were collected on the day of the neurobehavioural examination. The procedures for collecting and storing the blood samples are described elsewhere (Aarhus et al. 2018).

Glycated haemoglobin (HbA1c), serum levels of carbohydrate-deficient transferrin (s-CDT), thyroid-stimulating hormone (s-TSH) and free thyroxine (s-fT_4_) were analysed at UNILABS Medical Laboratory (Oslo, Norway). The method’s limit of detection for s-CDT was 0.4%, and a level of < 1.7% is considered normal by the laboratory (Bortolotti et al. 2006; Ellingsen et al. 2014; Bast-Pettersen et al., 2017). For HbA1c, the reference level given by the laboratory is 4.0–6.0%, the reference values for s-TSH are 0.40 to 4.0 mU/L, and s-fT4 < 22 pmol/L is considered normal.

The levels of cotinine, caffeine and nicotine in the serum were analysed at the National Institute of Occupational Health, and the procedure has previously been described (Aarhus et al. 2018).

### Work tasks and exposure

The subjects had worked in two workshop units of a construction company (Aarhus et al. 2018, 2019). The exposed workers at baseline worked as sheet metal workers performing grinding and welding (Fig. 1). This company was shut down in 1999, and most of the workers were offered jobs in other companies, which led to changes in work conditions for several of the subjects. Eight of the subjects originally unexposed to vibrating hand tools reported such exposure during the 22-year follow-up. Among the 27 subjects in the HAVS group, only two were not exposed during the follow-up time. Information about exposure, both work exposure and exposure in leisure time during the follow-up period, was obtained from an interview. The exposure was calculated as hours during the follow-up period. As the company had been shut down in 1999, we had no access to more accurate exposure parameters.

### Statistics

Continuous variables with a skewed distribution (skewness > 2) were log_10_ transformed. The log-transformed values were used in the statistical analysis, and the arithmetic means with standard deviations (SD) for these variables (hours of exposure, s-CDT and s-cotinine) are also presented in the tables). Hours with HAV exposure 1994–2017 comprised work exposure and leisure exposure, with the latter representing a small number.

To log transform the exposure values that were equal to zero exposure, their exposure values were set to 1 h. Student’s *t* tests were used to compare the 27 subjects with HAVS at baseline with the 13 subjects without HAVS at baseline, to compare the subjects according to their dichotomized levels of free thyroxine (s-fT_4_) and their dichotomized age, and to compare the Grooved Pegboard test results in 1994 according to HAVS status in 1994. Paired *t* tests were used to compare the pegboard performance in 1994 with the present performance and to compare postural tremor variables with rest tremor variables.

Multiple regression analysis was used to assess the associations between the predictors hours with HAV exposure 1994–2017, HAVS at baseline, and baseline Grooved Pegboard test results and the tremor parameters. Each predictor was analysed in a separate regression model which included the covariates age, s-cotinine, s-caffeine, HbA1c, s-CDT and s-fT4 (as continuous variables). To enhance power, we used multiple stepwise backward regression, and the independent variables were only included in the final models if they were retained by the backward selection procedure (Tables 3, 4, 5).

The dataset contained two missing values for the covariate HbA1c. To include the results of these subjects in the analysis, their values were estimated by imputation, and they were given the mean values of their respective age group.

The statistical analyses were performed with IBM SPSS^®^, version 22.0 (IBM Corporation, New York, USA). The level of significance was set at *p* ≤ 0.05.

## Results

Forty workers participated in the follow-up investigation. Table 1 shows their background and exposure data, the biomarker concentrations and their results of the Grooved Pegboard Test in the follow-up study by their HAVS status in 1994. Table 2 gives descriptions of the workers diagnosed with HAVS in 1994 (*n* = 27). Being diagnosed with HAVS at baseline did not predict increased tremor parameters at follow-up, while the co-predictors HbA1c and s-fT_4_ measured on the day of the examination influenced several of the Tremor Intensity parameters (Table 3).

**Table 1:** Background and exposure data for the 40 workers at follow-up by HAVS status in 1994

	HAVS at baseline *N* = 27		No HAVS at baseline *N* = 13		
	Arithmetic mean	SD	Arithmetic mean	SD	*p*
Age	60.2	10.4	60.7	10.5	ns
Self-reported exposure, hours with HAV exposure 1994–2017	3793	4738	769	1295	–
Log self-reported exposure hours with HAV exposure 1994–2017	3.27	0.54	1.78	1.5	0.004
Number of workers exposed during follow-up time	25		8		–
Prevalence of smokers/user of smokeless tobacco (%)	15	–	23		ns
s-nicotine (µg L^−1^)	5.456	12.43	9.539	16.05	ns
s-cotinine (µg L^−1^)^a^	103	227	104	176	–
Log s-cotinine^a^	0.30	1.34	0.45	1.43	ns
s-caffeine (µg L^−1^)	4542	2599	4106	2401	ns
Self-reported alcohol consumption (L/year)	3.7	4.3	2.8	3.1	ns
s-CDT^b^ (%)^a^	0.90	0.7	0.75	0.3	–
Log s-CDT^b^ (%)^a^	− 0.11	0.21	− 0.16	0.20	ns
HbA1c (%)^a^	5.6	0.7	5.9	1.0	–
Log HbA1c^a^	0.74	0.05	0.77	0.06	ns
s-fT_4_ pmol/L	14.0	1.7	13.9	2.1	ns
s-TSH mU/L	1.53	0.7	1.70	0.8	ns
Pegboard dominant hand	72.3	17.9	67.6^b^	10.3	ns
Pegboard non-dominant hand	80.4	20.6	75.0	16.3	ns

^a^Comparisons based on log-transformed values

^b^*N* = 12

**Table 2:** Descriptives for the workers diagnosed with HAVS in 1994 (*n* = 27)

	1994 study	2017 study
Numbness^a^/white finger attacks^b^/both, *n*	11/4/12	6/2/9
Stockholm Workshop Scale score (SD)	0.7 (0.8)	1.1 (1.1)
Self-reported hand numbness, *n* (%)	18 (67)	15 (56)

^a^During the last period of time, have you felt hand numbness? yes/no

^b^During the last period of time, have you felt white finger attacks? yes/no

**Table 3:** Associations between the predictor HAVS diagnosis in 1994 and Tremor Intensity in 2017 among the 40 male workers, dominant hand

Predictors/covariates	Postural tremor				Rest tremor			
	Postural tremor, fully adjusted regression coefficients		Postural tremor, Final model		Rest tremor, fully adjusted regression coefficients		Rest tremor, Final model	
		*p*		*p*		*p*		*p*
Age	0.001	0.26	–		0.001	0.07	–	
HAVS at baseline	− 0.010	0.54	–	–	0.12	0.39	–	
s-cotinine^a^	0.12	0.035	0.011	0.04	0.003	0.49	–	
s-caffeine	5.401E−7 (~)	0.87			− 2.410E−6 (~)	0.36	–	
HbA1c^a^	0.497	0.008	0.682	< 0.001	− 0.089	0.54	–	
s-CDT^a^	0.023	0.55	−	−	6.874E−5 (~)	0.99	−	–
s-fT4	0.008	0.12		−	0.014	0.001	0.011	0.002

Estimates in the final model based on multiple stepwise backward regression, mutually adjusted for the other covariates shown in the table. HAVS at baseline: not in the final model

^a^Log-transformed values used for comparisons

The exposure during the follow-up time calculated as “hours with HAV exposure 1994–2017”, was not associated with increased tremor (Table 4).

**Table 4:** HAV exposure (h) during the 22-year follow-up (hours with HAV exposure 1994–2017) and Tremor Intensity in 2017 among the 40 male workers, dominant hand

Predictors/covariates	Postural tremor				Rest tremor			
	Postural tremor, fully adjusted regression coefficients		Postural tremor, Final model		Rest tremor, fully adjusted regression coefficients		Rest tremor, Final model	
		*p*		*p*		*p*		*p*
Age	0.001	0.28	–		0.001	0.07	–	
HAV exposure during the 22-year follow-up (hours with HAV exposure 1994–2017)	− 0.002	0.24	–		− 0.001	0.33	–	
s-cotinine^a^	0.011	0.054	0.011	0.0042	0.002	0.66	–	
s-caffeine	6.4442E−7	0.84	–		− 2.052E−6	0.43	–	
HbA1c^a^	0.517	0.004	0.682	< 0.001	− 0.126	0.37	–	
s-CDT^a^	0.024	0.53	–		0.009	0.77	–	
s-fT4	0.008	0.10	–		0.015	0.001	0.011	0.002

Dominant hand. The final model based on multiple stepwise backward regression, mutually adjusted for the other covariates shown in the table

^a^Log-transformed values used for comparisons

The Grooved Pegboard test results at baseline were significantly associated with the Tremor Intensity outcomes in the final model (Table 5).

**Table 5:** Associations between the predictor Grooved Pegboard test results at baseline (1994) and Tremor Intensity in 2017 among the 40 male workers, dominant hand

	Postural tremor				Rest tremor			
	Postural tremor, fully adjusted regression coefficients		Postural tremor, Final model		Rest tremor, fully adjusted regression coefficients		Rest tremor, Final model	
	*p*		*p*		* p*		*p*	
Age	3.271–5 (~)	0.97	–	–	0.001	0.45	–	
Pegboard at baseline dominant hand	0.001	0.066	0.001	0.02	0.001	0.075	0.001	0.016
s-cotinine^a^	0.13	0.025	0.013	0.02	0.003	0.50	–	
s-caffeine	1.538E−7 (~)	0.96			− 2.407E−6 (~)	0.34	–	
HbA1c^a^	0.621	0.001	0.703	0.001	− 0.045	0.75	–	
s-CDT^a^	0.25	0.49	–		0.10	0.72	–	
s-fT4	0.005	0.30	–		0.12	0.004	0.01	0.003

The final model based on multiple stepwise backward regression, mutually adjusted for the other covariates shown in the table

^a^Log-transformed values used for comparisons

All subjects had free thyroxine (s-fT_4_) levels lower than the reference limit of 22 pmol/L. The median value was 15 pmol/L, with 16 subjects having values ≥ 15 pmol/L. For the dominant hand, the postural Tremor intensity test results were 0.18 (SD: 0.08) vs 0.13 (SD: 0.03) (*p* = 0.02), for the group with s-fT_4_ values ≥ 15 pmol/L vs those with lower values, respectively. The same trend was observed for rest tremor; in the dominant hand, the Tremor Intensity was 0.10 (SD: 0.05) vs 0.06 (SD: 0.02) (*p* = 0.006), respectively (not tabulated).

Neither alcohol consumption nor age were significantly associated with increased tremor. The twenty subjects older than 60 years had a nonsignificantly higher postural Tremor Intensity than the younger subjects; for the dominant hand, these values were 0.16 vs 0.13 (*p* = 0.12). For the rest tremor, the differences were even smaller; in the dominant hand, 0.08 vs 0.07 (*p* = 0.27) in those older than 60 years vs those who were younger, respectively (not tabulated).

**Fig. 2:**
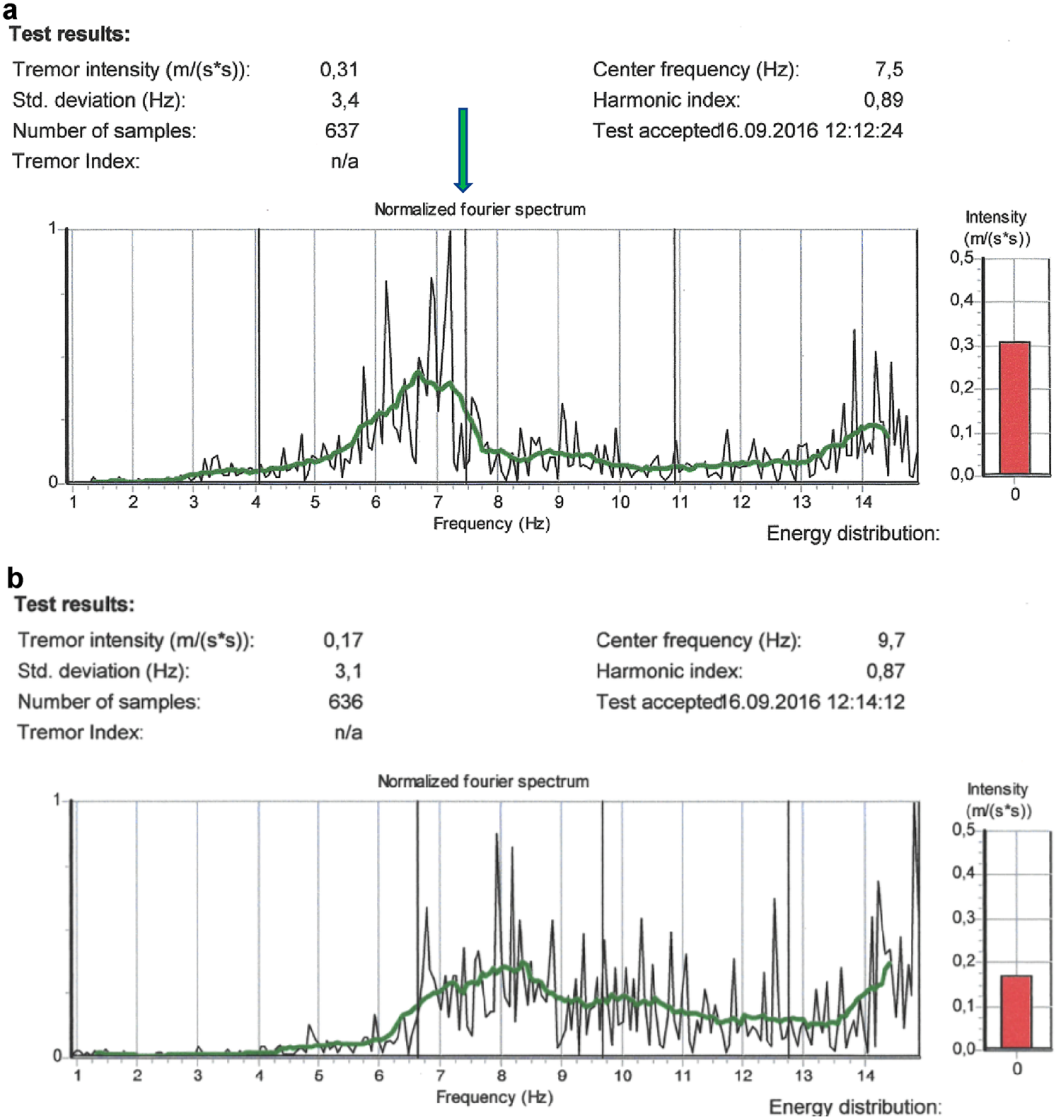
**a** Postural tremor. **b** Rest tremor. **a**, **b** Postural vs rest tremor. The same subject (aged 75 years) was tested with two activation conditions. Postural tremor: higher Tremor Intensity and lower Tremor frequency than when the subject rested his arm on the table

Table 6 shows the comparison between postural tremor and rest tremor parameters. As expected, the magnitude of the tremor, Tremor Intensity, was lower when the subjects placed their hands in a resting position. The Center Frequency significantly increased from 7.4 Hz to 9.8 Hz for the dominant hand in the resting position. Figure 2 gives an illustration of a typical power spectrum in one subject, showing that the tremor spectrum was shifted towards a higher frequency.

**Table 6:** Comparison between tremor measures for all subjects from two different activation conditions, postural tremor vs rest tremor

	Postural tremor (*N* = 40)		Rest tremor (*N* = 40)		*p*
	Arithmetic mean	SD	Arithmetic mean	SD	
Dominant hand					
Tremor Intensity (ms^−2^)	0.15	0.06	0.08	0.04	< 0.001
Center Frequency (Hz)	7.4	1.3	9.8	1.3	< 0.001
Frequency Dispersion (Hz)	3.1	1.1	3.1	1.0	0.99

## Discussion

In this 22-year cohort study of workers exposed to HAV, self-reported cumulative exposure calculated as hours with HAV exposure 1994–2017, during the follow-up time was not associated with increased tremor, and HAVS at baseline did not predict increased tremor 22 years later. A test for manual dexterity (the Grooved Pegboard Test) predicted the magnitude of tremor, the Tremor Intensity 22 years later. While alcohol consumption was not associated with the tremor parameters in this group of low consumers, medical conditions such as dysregulated glucose metabolism (HbA1c) and free thyroxine s-fT_4_ levels were associated with increased tremor. The use of nicotine-containing products also affected postural tremor to a limited degree. The activation conditions had a significant effect on the tremor parameters. No significant age effects were observed.

The CATSYS test system has been used in several studies to evaluate the effects on the central nervous system following exposure to neurotoxins, but has only been used in two published studies of tremor in HAV-exposed subjects (Edlund et al. 2015; Bast-Pettersen et al. 2017).

## Tremor related to hand-arm-vibration exposure during the 22-year follow-up

We found no associations between exposure to HAV during the follow-up time calculated as hours with HAV exposure 1994–2017, and the magnitude of tremor assessed as Tremor Intensity. In a previous study of workers exposed to vibrating hand tools, cumulative exposure to HAV was statistically significantly and positively associated with increased postural and rest tremor among smokers and users of smokeless tobacco (Bast-Pettersen et al. 2017). In the present study, only 18% were smokers, and their current concentrations of s-cotinine were substantially lower than in the study by Bast-Pettersen et al. (2017). Our findings appear to be in line with the findings among non-smokers in the Bast-Pettersen and coworkers (2017) study. No age effects on tremor measures were found in the present study. This was slightly unexpected, since the age range was 44–77 years, with 25 of the subjects being in the age group of 50–70 years, and eight subjects were older than 70 years.

## Tremor related to HAVS at baseline

Our study included 27 workers with HAVS symptoms in 1994. As to the long-term change in HAVS symptoms, our prior studies showed no statistically significant change in Stockholm Workshop Scale score from 1994 to 2017, neither for hand numbness or for white finger attacs (Aarhus et al. 2018, 2019). The present study, assessing later tremor, showed no association between HAVS symptoms in 1994 and Tremor in 2017. During follow-up, 8 out of 13 subjects in the initial non-HAVS group crossed over to HAV-exposed work. This could result in an underestimate of the association. However, only 2 out of these 8 persons reported symptoms of HAVS in 2017, and we do not believe that the statistically non-significant association between HAVS in 1994 and tremor in 2017 is due to this crossover. The finding that HAVS at baseline did not predict increased tremor at follow-up is in contrast to our previous study of road maintenance workers (Bast-Pettersen et al. 2017). Based on the findings in that study, we suggested that tremor might be a part of the clinical picture of HAVS. Edlund et al. (2015), in their follow-up study, did not find increased tremor in HAV-exposed subjects. In their study, increased age and nicotine use appeared to be the strongest predictors of tremor.

A difference between the present study and our previous study of road maintenance workers (Bast-Pettersen et al. 2017) was that in the 2017 study, we studied workers with ongoing exposure, while the present study included previously exposed workers. This might indicate that our findings in the 2017 study were due to a kind of acute effect. Our findings in the present study of mostly previously exposed workers could, therefore, indicate that tremor is not expected to be a part of the long-term clinical picture of HAVS, at least not in subjects with low tobacco consumption. Future studies are required to address this question.

## Tremor related to manual dexterity at baseline

As previously mentioned, the 1998 consensus statement (Deuschl et al. 1998) recommended including measures of performance, for instance, a pegboard test, in tremor study designs, and our study included such a test. In contrast to the finding that HAVS at baseline did not affect the tremor parameters, the pegboard test results at baseline were significantly associated with all the Tremor Intensity outcomes.

In the baseline assessment, the results of the Grooved Pegboard Test were closely related to the assessment of HAVS status at baseline. Analyses of all the workers in the 1994 study who fulfilled our inclusion criteria (*n* = 110) showed that the original HAVS group (*n* = 68) took 5.6 s (95% CI: 1.5–9.8) longer to complete the Grooved Pegboard Test in 1994 than the workers without HAVS (*n* = 42), while the age-adjusted estimate was 4.3 s (95% CI: 0.3–8.3). The same trend was found for the workers who were enrolled in the follow-up study (*n* = 40). The workers with HAVS in 1994 (*n* = 27) took 9.1 s (95% CI 1.7–16.5) longer to complete the Grooved Pegboard Test in 1994 than the workers without HAVS (*n* = 13), and age did not confound this relationship. This might suggest the possibility of an association of a neuro-sensory component of HAVS at baseline and tremor at follow-up.

The deterioration in the pegboard performance over the 22-year follow-up period was 6.3 and 9.3 s for the dominant and non-dominant hand, respectively (*p* < 0.001). This deterioration was highly statistically significant, but the performance was normal for their age group in each wave of testing, according to published norms (Heaton et al. 2004). This indicates that their hand function was quite stable over the 22 years and that their impairment was a result of normal ageing.

In a study of young divers that were tested three times during a 12-year span, no impairments in this test were found over this 12-year time period (Bast-Pettersen et al. 2015). In that study, the oldest divers were under 45 years at the last examination, indicating that the age effect on manual dexterity, at least measured with the Grooved Pegboard Test, appears later than 45 years of age.

## Tremor related to current medical conditions and life-style habits (tobacco and alcohol consumption)

To our knowledge, only one published study of tremor in vibration-exposed workers has assessed consumption of alcohol, nicotine and caffeine using biomarkers of exposure rather than by self-report (Bast-Pettersen et al. 2017).

Cotinine has a half-life of ~ 16 h compared to nicotine’s 2-h half-life, and cotinine values are better estimates of current nicotine use in the final hours prior to the serum sampling (Hukkanen et al. 2005). Only seven subjects reported that they were smokers in the follow-up study, four (15%) of the subjects with HAVS at baseline and three (23%) of the non-HAVS subjects. In the regression analyses, s-cotinine was used instead of self-report, and s-cotinine was associated with increased postural tremor in the dominant hand. The finding of increased tremor among smokers has previously been found when smoking was assessed by self-report (Bast-Pettersen et al. 2004, 2005) and with biological markers (Ellingsen et al. 2006; Bast-Pettersen et al. 2017). Even in the present study, where most subjects were low consumers, smoking habits as assessed with s-cotinine, was associated with increased tremor.

The subjects’ self-reported alcohol consumption was 3.4 L alcohol/year. This is in accordance with the figures for self-reported alcohol consumption in 2017, provided by the Norwegian Institute of Public Health (Bye 2018). Based on a similar questionnaire, they reported an alcohol consumption equal to 3.8 L alcohol/year for the age group of 55–64 years. In the present study, the plasma concentration of s-CDT was used in the analyses instead of the self-reported alcohol consumption. We found no associations between the s-CDT levels and the tremor parameters. Because the average s-CDT value of 0.85% was well below the upper reference limit of the laboratory (1.7%), an influence of heavy drinking on the results could not be expected in the present study.

It is of interest that dysregulated glucose metabolism (HbA1c) and free thyroxine s-fT_4_ levels were associated with several of the tremor parameters. From a clinical point of view, it could be expected that higher levels of s-fT_4_ would have an association with increased tremor, but few studies have documented this association with a quantitatively based tremor test (Milanov and Sheinkova 2000). In overt hyperthyroidism, elevated levels of the thyroid hormones (thyroxine and triiodothyronine) may enhance physiological tremor producing a low-amplitude postural tremor of relatively high frequency (about 7 Hz) (Elble 2009). In the present study, the free thyroxine levels were all lower than the reference limit value (normal range: s-fT4 10–22 pmol/L), and an association between tremor and levels of s-fT4 in the normal range has, to our knowledge, not been previously reported.

HbA1c is a good measure for detecting dysregulated glucose metabolism, before the diagnosis is clinical evident. Moreover, diabetic polyneuropathy is common and may precede other clinical symptoms of diabetes, and peripheral neuropathies of different origins may be associated with tremor. Tremor, mostly postural, has been described in patients with peripheral neuropathies of different origins (Fahn and Jancovic 2007; Elble 2009; Wasielewska et al. 2013).

## Activation condition–comparison between postural tremor and rest tremor

As expected, we found a higher magnitude of the postural tremor than the rest tremor when the subject’s arm was supported against gravity. In addition, we found that the rest tremor had a higher Center Frequency than the postural tremor (Table 6), and this is in accordance with what we found in a study of road maintenance workers (Bast-Pettersen et al. 2017). Our finding confirms the finding of the 2017 study (Bast-Pettersen et al. 2017) that the activation condition is of importance and has a large impact on tremor parameters and must be taken into account when testing tremor.

### Aspects of validity, strengths and limitations

The long follow-up time is a major strength of this study. The relatively high participation rate (66%) of the invited subjects indicates that the findings in the present study are representative of the original group studied in 1994. A major strength is that tremor was measured with a standardized tremor test. Other strengths of the study are that potential confounders such as alcohol consumption, use of nicotine products, consumption of caffeine-containing drinks and current medical conditions such as dysregulated glucose metabolism and free thyroxine levels were assessed using biomarkers rather than assessment by self-report. All the participants were males; therefore, there were no potential sex effects on the tremor results, which could potentially confound associations between exposure and outcomes*.*

The relatively low number of 40 subjects could represent a weakness. In the statistical analyses, the final regression models included three predictors at maximum. A major weakness of the study is that exposure to vibration during follow-up was assessed by self-report. As the company had been shut down, we had no access to more accurate exposure parameters.

## Conclusions

HAV exposure during the 22-year follow-up was not associated with increased tremor.

HAVS in 1994 did not predict increased tremor 22 years later.

The test for manual dexterity at baseline, the Grooved Pegboard test results, were associated with increased tremor 22 years later.

Current medical conditions and life-style habits, including dysregulated glucose metabolism (HbA1c), free thyroxine s-fT_4_ levels and smoking, assessed with s-cotinine, were associated with increased tremor.

Activation conditions (e.g., hand position) are important when testing tremor.
